# Littre’s Hernia Incidentally Found During Laparoscopic Indirect Inguinal Hernia Repair in a Child

**DOI:** 10.7759/cureus.66528

**Published:** 2024-08-09

**Authors:** Ryo Tsukada, Shun Iwasaki, Souji Ibuka, Ryuta Saka

**Affiliations:** 1 Department of Pediatric Surgery, National Hospital Organization Fukuyama Medical Center, Fukuyama, JPN

**Keywords:** ileal resection, child, laparoscopy, littre’s hernia, meckel’s diverticulum

## Abstract

Littre's hernia is a rare type of hernia in which Meckel’s diverticulum is found in the hernia sac. While most cases present with incarceration, incidentally discovered cases of Littre's hernia without incarceration are even rarer. A three-month-old boy was referred to our hospital with a three-month history of right inguinal swelling. Although the swelling was reducible, re-prolapse was readily observed. Small bowel obstruction and gastrointestinal bleeding had not been experienced. Laparoscopic herniorrhaphy was planned for right inguinal hernia repair at four months of age during which Littre’s hernia was incidentally discovered intraoperatively. Following laparoscopic herniorrhaphy, extracorporeal small bowel resection was performed. The postoperative course was uneventful and the patient was followed up for nine months without symptoms. Laparoscopic herniorrhaphy is a useful surgical technique, as it may facilitate the detection of unexpected complications, which might be overlooked with the inguinal approach.

## Introduction

Meckel’s diverticulum (MD) is the most common congenital gastrointestinal anomaly. Although MD is often asymptomatic, it is known that children are more likely to be symptomatic than adults [1]. MD may manifest as intestinal obstruction, gastrointestinal bleeding, acute intraabdominal inflammation, umbilical anomalies, and Littre’s hernia (LH). When MD protrudes into any hernia sac, it is called Littre's hernia (LH) [2]. LH is a rare presentation of MD and represents less than 1% of MD cases [3]. Herein, we report a pediatric case of LH treated by ileal resection following laparoscopic herniorrhaphy.

## Case presentation

**Figure 1:**
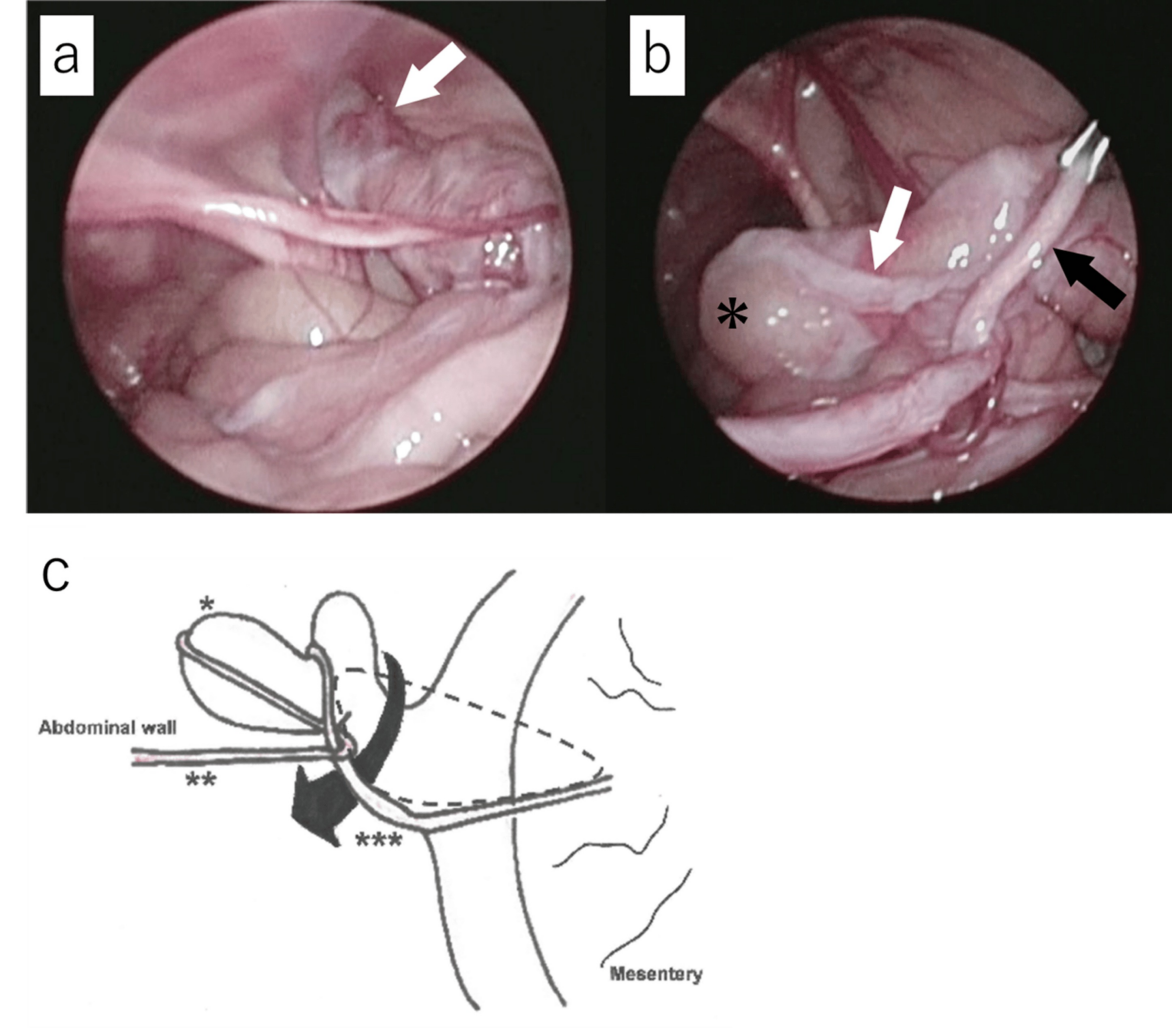
Intraoperative findings and a schematic illustration (a) Meckel’s diverticulum (white arrows) protruded into the right processus vaginalis. (b) The remnant of the vitelline duct (white arrow) including Meckel's diverticulum (asterisk) and the mesodiverticular band (black arrow) were entwined. (c) A schematic illustration of (b). Entanglement of the remnant of the vitelline duct and the mesodiverticular band was confirmed laparoscopically. The tip of Meckel's diverticulum was herniated through the hernia orifice surrounded by the mesodiverticular band and the base of Meckel's diverticulum itself (single asterisk: Meckel's diverticulum, double asterisk: the remnant of the vitelline duct, triple asterisk: the mesodiverticular band, dashed area: the hernia orifice). Additionally, the tip of Meckel's diverticulum was herniated into the right inguinal canal (Litrre's hernia). Credit for (c): The authors

A three-month-old boy was referred to our hospital with a three-month history of right inguinal swelling. Physical examination revealed a soft swelling in the right inguinoscrotal region. Although the swelling was reducible, re-prolapse was readily observed. Ultrasonography revealed protruded intestines. Small bowel obstruction and gastrointestinal bleeding had not been experienced, and the appearance of the umbilicus was normal. Elective laparoscopic herniorrhaphy (laparoscopic percutaneous extraperitoneal closure) was planned for right inguinal hernia repair at four months of age [4]. A 3-mm cannula for laparoscopy was placed through an umbilical incision under general anesthesia. Following the establishment of pneumoperitoneum with CO_2_ insufflation, a herniated Meckel’s diverticulum (MD) was confirmed (Figure 1a). Successful reduction of the MD was achieved by manual compression. The remnant of the vitelline duct and the mesodiverticular band were also detected, and the two structures were entwined (Figure 1b). MD was delivered through a slightly extended umbilical wound. This positioning occurred secondary to an internal hernia of the distal part of the MD through the hernia orifice consisting of the mesodiverticular band and proximal MD, although the MD did not appear to be significantly twisted (Figure 1c).

**Figure 2:**
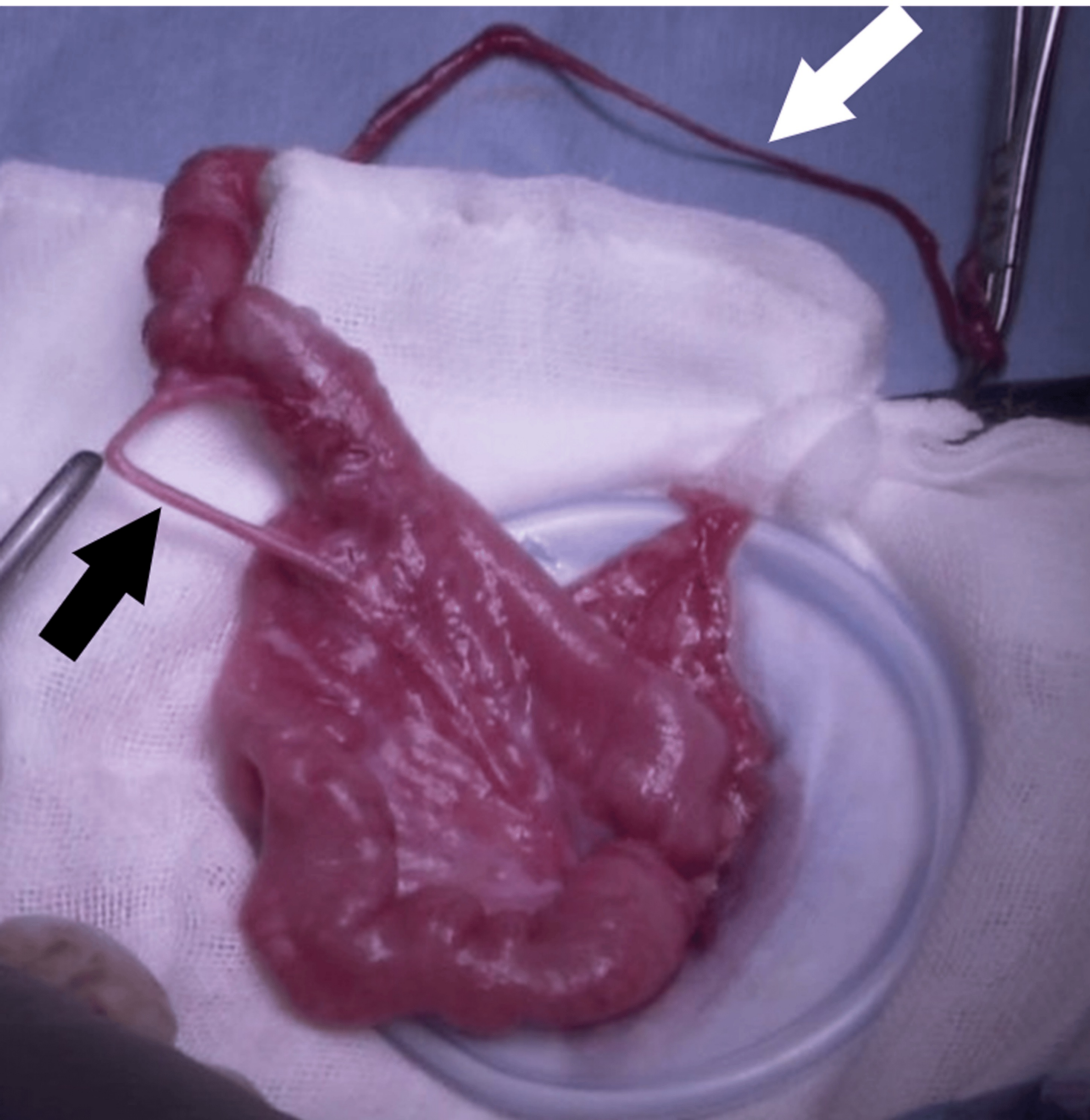
Intraoperative photograph Meckel's diverticulum was delivered through a slightly extended umbilical wound (the white arrows indicate the remnant of the vitelline duct and the black arrows indicate the mesodiverticular band).

Following laparoscopic herniorrhaphy, extracorporeal small bowel resection was performed through an umbilical incision extended to 2 cm in length (Figure 2).

The remnant of the vitelline duct terminated at the level of the abdominal wall and was completely resected. Bowel continuity was restored in an end-to-end fashion. The patient's postoperative course was uneventful, and histopathological examination revealed ectopic gastric mucosa in the MD. There was no evidence of right inguinal hernia recurrence or small bowel obstruction at the nine-month follow-up.

## Discussion

MD results from incomplete obliteration of the omphalomesenteric (vitelline) duct, which connects the yolk sac and embryonal midgut during early fetal development. MD is known as the most common gastrointestinal congenital anomaly, which occurs in 0.3-2.9% of the general population [5]. Although symptomatic MD manifests as bleeding, intussusception, volvulus, internal hernia, and inflammation, protrusion into a hernia sac (Littre’s hernia) is relatively rare. In adults, femoral hernias are the most frequent cause of Littre’s hernia (LH), followed by inguinal hernias and umbilical hernias [6]. Conversely, inguinal and umbilical hernias are more prevalent in childhood [7].

 We reviewed well-documented case reports of pediatric (age ≤ 18 years old) LH associated with indirect inguinal hernias (Table 1).

**Table 1:** Pediatric (age < 18 years old) cases of Littre's hernia in indirect inguinal hernia M=Male, F=Female, L=Left sided, R=Right sided, NA=Not available

Case	Year	Author	Age	Sex	Laterality	Incarceration	Perforation	Approach	Postoperative course
1	1954	Charles E [7]	3 weeks	M	R	(＋)	(－)	Inguinal	Uneventful
2	1958	Kline AH [8]	4 weeks	M	R	(＋)	(－)	Abdominal	Uneventful
3	1959	Baillie RC [9]	1 month	M	R	(＋)	(－)	Inguinal	Uneventful
4	1974	Krausz M et al. [10]	1 month	M	L	(＋)	(－)	Inguinal	Uneventful
5	1982	Mishalany HG et al. [11]	10 months	M	R	(＋)	(－)	Inguinal	Uneventful
6	1989	K. Ravikumar et al. [12]	8 months	M	R	(－)	(－)	Inguinal	Uneventful
7	2005	Messina M et al. [2]	16 days	M	R	(＋)	(－)	Inguinal	Uneventful
8	2005	Messina M et al. [2]	12 days	M	NA	(＋)	(－)	Inguinal	Uneventful
9	2006	Vaos G [13]	2 years	M	R	(＋)	(－)	Inguinal	Uneventful
10	2008	Akin M et al. [14]	7 years	M	R	(＋)	(－)	Inguinal	Uneventful
11	2008	Chan KW et al. [15]	11 months	M	L	(－)	(－)	Laparoscopic	Uneventful
12	2008	Chan KW et al. [15]	3 years	M	R	(＋)	(－)	Laparoscopic	Uneventful
13	2011	Pampal A et al. [16]	3 years	M	R	(＋)	(－)	Inguinal	Uneventful
14	2013	S. Visnjic et al. [17]	4 months	M	R	(＋)	(－)	Abdominal	Uneventful
15	2014	Qin D et al. [18]	4 months	M	L	(＋)	(－)	Inguinal	Uneventful
16	2014	Singh RR et al. [19]	2 years	M	R	(＋)	(－)	Inguinal	Uneventful
17	2016	Magagi IA et al. [20]	18 years	M	R	(＋)	(－)	Inguinal	NA
18	2018	Bakal U et al. [3]	2 months	M	R	(＋)	(－)	Abdominal	Uneventful
19	2018	Bakal U et al. [3]	15 months	F	L	(＋)	(－)	Abdominal	Wound infection
20	2018	Bakal U et al. [3]	54 months	M	R	(－)	(－)	Inguinal	Uneventful
21	2018	Bakal U et al. [3]	1 month	M	R	(＋)	(－)	Inguinal	Uneventful
22	2019	Velásquez-Bueso AE et al. [21]	6 years	M	R	NA	(－)	Inguinal	NA
23	2020	Usman A et al. [22]	16 years	M	L	(＋)	(－)	Inguinal	Uneventful
24	2021	Gawrieh B et al. [23]	3 years	M	L	(＋)	(－)	Inguinal	Uneventful
25	2022	Gupta R et al. [24]	27 days	M	R	(＋)	(＋)	Abdominal	Uneventful
26	2023	Odongo CN et al. [25]	3 years	M	L	(－)	(＋)	Inguinal	Uneventful
27	2023	Presented case	5 months	M	R	(－)	(－)	Laparoscopic	Uneventful

The median age at diagnosis was 8 months (range: 12 days to 18 years), and 15 patients (55.5%) were under 1 year of age. Most patients were male (96.2%), and indirect inguinal hernias were predominantly found on the right side (70.3%). The postoperative courses of all patients were uneventful, except for one case in which the patient developed a wound infection. 

 Our review showed no accurate preoperatively diagnosed cases. Preoperative identification of MD within a hernia sac is difficult [22,26,27]. There are often no characteristic diagnostic clinical symptoms to differentiate LH from other hernias. LH repair consists of resection of the MD and herniorrhaphy. Complete excision of the MD and herniorrhaphy were simultaneously performed in 26 out of 27 cases. Only three cases (including our case) were performed laparoscopically. In this review, 22 out of 26 cases (84.6%) had incarceration.

In our case, the inguinal hernia was reducible, and specific symptoms of MD, including gastrointestinal bleeding and small bowel obstruction, were absent. Therefore, the patient was planned for an elective surgery. Despite the internal hernia and the presence of the MD within the hernia sac, small bowel obstruction was not observed. There are few reports of incidental LH discovered during laparoscopic herniorrhaphy. Chan et al. reported two cases treated via a laparoscopic approach, one of which had complications related to intestinal perforation that occurred during the reduction of the herniated MD [16]. In cases of incarceration, it is important to reduce the intestines more carefully.

Since most published cases were incarcerated cases, we speculated that there may be cases of overlooked LH when the inguinal approach is employed in reducible cases. Laparoscopic herniorrhaphy may reveal ‘hidden’ LH, which might be overlooked when using the inguinal approach.

## Conclusions

Herein, we report a case of incidentally observed LH during laparoscopic herniorrhaphy. Laparoscopic herniorrhaphy is a useful surgical technique because it may be able to detect unexpected complications, which might be overlooked when using the inguinal approach.
